# Advocacy resource: engaging the media and promoting your cancer program in Africa

**DOI:** 10.1186/1750-9378-8-S1-S5

**Published:** 2013-07-15

**Authors:** R Renee Reams, Folakemi T Odedina, Shannon Pressey

**Affiliations:** 1 College of Pharmacy & Pharmaceutical Sciences, Florida A&M University, Tallahassee, Florida 32307, USA; 2 College of Pharmacy, University of Florida, Gainesville, Florida, 33772, USA

## Abstract

To address the need for a significant increase in cancer advocacy programs in Africa, the University of Florida (UF), the Prostate Net, and the African Organization for Research and Training in Cancer (AORTIC) co-hosted the first biennial *International Workshop on Cancer Advocacy for African Countries* (*CAAC*) on November 29, 2011, one-day prior to AORTIC’s 8^th^ International Cancer Conference in Cairo, Egypt. Over 70 African cancer advocates representing about 12 African countries participated in this workshop.The primary goal of the one-day workshop was to inform, educate and empower African cancer advocates to increase the promotion of their cancer programs. The first half of the workshop consisted of five formal PowerPoint presentations focused on the following topics: (a) Understanding Your Community and Assessing your Community Health Assets and Needs; (b) Developing a successful advocacy model for your cancer program; (c) Developing a Relationship with your Elected Officials to Advocate Cancer-related Policies; (d) Engaging the Media and promoting your cancer program; and (e) Developing advocacy plans for sustainability. In this article we summarize the informational content given in the PowerPoint presentation entitled *“Engaging the Media and promoting your cancer program”.* The content given in this article is useful as a how-to guide for both the beginner and the experienced cancer advocate who wants to establish/promote a cancer awareness program.

## Introduction

Reaching out to a population of people to raise awareness about cancer incidence, mortality rates and cancer prevention is a challenge under any circumstance; but when the population resides on the continent of Africa, where most of Africa’s 2,000 languages have no word for cancer, promoting cancer advocacy is a very daunting task. However, a look at recent cancer statistics which estimated one million new cancer cases in sub-Saharan Africa by 2012, also predicted that this number would double to 2 million per year by 2024, and would result in more than 0.5 million annual deaths from cancer in Africa [1]. These figures alone support the dire need for increased cancer advocacy in Africa. Cancer advocacy is very limited in Africa due to the intense focus on infectious diseases, in Africa for the past six decades. Ironically, the growing increase in cancer in Africa is thought to be a possible complication of long-term HIV infection, both in low-resource countries and in resource-rich countries [2,3]. Several reports point to the fact that long-term HIV infection may also predispose an individual to infectious disease-related cancers e.g. liver, cervical, lymphomas and Kaposi sarcoma [4]. The traditional AIDS-defining cancers, which include Kaposi sarcoma, cervical cancer, and non-Hodgkin lymphoma are common comorbidities that afflict HIV-positive individuals [2]. Additionally, there are several non-AIDS-defining cancers such as Hodgkin lymphoma, hepatocellular carcinoma, and lung cancer that have increased in incidence in resource-limited regions. Taken together, cancer statistics along with the knowledge that long term HIV infection is contributing to the growing problem of cancer in Africa, lend strong support for increasing cancer advocacy in Africa.

Using various forms of existing media in Africa can help move cancer advocacy from a non-priority to a priority status in African countries. The *1st International Workshop on Cancer Advocacy for African Countries* brought African cancer advocates from across the continent of Africa together to listen, learn and dialog on the need to spread cancer advocacy in Africa [5]. Over 70 African cancer advocates participated in this workshop, with approximately 20 cancer advocates receiving travel awards through funding support from the United States National Cancer Institute. The primary goal of the workshop was to empower beginners and experienced cancer advocates to spread knowledge and raise public awareness about cancer, cancer prevention and early detection. Invited speakers presented six workshop topics: **(a)** Understanding Your Community and Assessing your Community Health Assets and Needs; **(b)** Developing a successful advocacy model for your program; **(c)** Developing a Relationship with your Elected Officials to Advocate Cancer-related Policies; **(d)** Engaging the Media and promoting your cancer program; and **(e)** Developing advocacy plans for sustainability. In this article, a summary of the media presentation is given as a list of steps to promote cancer advocacy. Below are details of six sequential steps for engaging the media when initiating and promoting African cancer advocacy programs.

### Step 1. Decide on your purpose

It is essential that a purpose and target audience is defined before initiating cancer advocacy activities.

To decide your purpose, answer the question: What are the major public healths issues concerning the cancer you want to raise awareness about? Which issues has causes that can bring about the most impact for populations/communities affected? For example: Do you and the affected community want to educate the public about incidence, mortality, family history? Do you want to promote screening? Do you want to focus on men’s health, women’s health or children’s health? Do you want to promote prostate screening? [6] Are you *interested in the prevalence of* HPV in women in your communities? [7][8] Do you want to develop a “call to action” plan such as fighting cancer with cell phones as done in the Kilimanjaro Cervical Cancer Screening Projects aimed at reaching women in remote villages? [9]

### Step 2. Recruit members

Identify, involve and recruit community members affected by the cancer(s) you want to promote in order to develop the manpower needed to move your cancer advocacy programs forward.

Deciding whom to recruit as members, should start with getting to know the affected communities, including the members’ needs and wants. You will need community buy-in and involvement to be an effective advocacy group. It is highly recommended that the purpose and ultimate goals of your cancer advocacy group focus on community needs. If it does, recruitment of members will be a natural progression. It is also important to educate and train your members as they are recruited rather than waiting until you get a sizable membership to do the training. This will strengthen your cancer advocacy program and provide seasoned members to educate and train newly recruited members. Once recruited, educated and trained, members should decide among themselves the leadership roles they will assume; this empowers them to promote and lead cancer advocacy activities in the community. Survivors, families of survivors, health care professionals, politicians, journalists, non-survivors are all excellent potential members.

### Step 3. Brand your program

Once you have solidified your cancer advocacy purpose and recruited members, create a unique logo and name that lets everyone know at a glance who you are and what you are advocating. ***Some examples of branded programs are described below.***

**• Tunde and friends foundation;** founded in Nigeria in May 1995 by Tunde Akinremi, a Nigerian Air Force Pilot left quadriplegic after an auto accident. Tunde’s purpose was to reach out to individuals and families shattered by effects of disabilities. To date, with the expert help of his wife, Dr. Titilola Akinremi (a trained pathologist), Tunde and Friends has included Prostate Cancer Awareness and an annual health screening and health fair as part of their community/village outreach efforts.

• Tanzania 50 plus campaign - prostate health education

Rev. Canon Dr. Emmanuel J. Kandusi is founder and Chair of the Tanzania 50 Plus Campaign. This Advocacy Group focuses on prostate cancer literacy, advocacy and support initiatives, and helps others to start up advocacy programs.

• The Prostate Net.

There was little to no readily accessible information to help prostate cancer patients and their families make informed treatment decisions when Mr. Virgil Simmons was diagnosed with prostate cancer so he founded the Prostate Net, 16 years ago. The Prostate Net is a non-profit patient education and advocacy organization. Using the experiences gained as marketing professional and as a 16-year survivor of prostate cancer and a patient advocate, Virgil Simmons built an international organization that uses a matrix of informational techniques (Web site, 800#, email and personal team counselors, public forums, newsletters and community disease interventions) to address disease risk awareness and early disease detection.

**• Alachua County Prostate Health Outreach Program (PHOP)**.

Initiated by Dr. Folakemi Odedina (Professor of UF College of Pharmacy and Associate Director of the UF Shands Cancer Center) in Gainesville, Florida, USA, the mission of the Florida Prostate Health Outreach Program (PHOP) is to reduce the morbidity and mortality of prostate cancer in the State of Florida. To achieve this mission, three services are offered through PHOP: (i) Community Relations to educate and empower underserved communities; (ii) Organizations / Providers Relations to facilitate state-wide collaborative efforts among community-based cancer organizations, providers, public and private organizations, and academic institutions; and (iii) Research Relations to foster community-based participatory research (CBPR).

• Caribbean support group/ US TOO, Bahamas:

A prostate cancer support group in The Bahamas, the US TOO Chapter was started by Dr. Robin Roberts, prostate cancer survivors and concerned citizens of Nassau, Bahamas. US TOO is part of an International Prostate Cancer Education and Support Network and The Bahamas chapter provides prostate cancer education and screening annually.

### Step 4. Select partners and stakeholders

Let others help you raise awareness about your cancer program. Prepare a 3 minute script that gives the highlights of your cancer program and how a desired partner or stakeholder can contribute favorably to your cause. Then make appointments to see potential partners and stakeholders, quickly give them the facts and tell them why you need them as a partner. Below are examples of potential partners in Africa.

**• Ministers of Health or Government Health Official.** Write letters to your ministers of health informing them of the issues, cancer facts and statistics surrounding your cancer program. If possible, show statistics or medical reports that shows them the degree that the districts or regions they serve are impacted by a cancer mortality or prevalence. Ask your government officials to attend your next health fair, cancer screening or visit a village or community. Having a minister of health to meet and greet participants at your cancer advocacy activity will further confirm cancer as a top priority in Africa.

**• Survivors.** It is imperative to always recruit survivors as part of your cancer advocacy outreach programs. Invite survivors to share their stories at community events. If possible and with their permissions, video their testimonials and share at future events.

**• HealthCare professionals at hospitals/clinics.** Make sure to recruit nurses, pharmacists, doctors, surgeons, on your team. Also consider inviting research scientists to join your team so that they can share what they are doing in the laboratory to fight and cure cancer. Make sure you leave time for the community to ask questions and dialogue with partners and stakeholders.

### Step 5. Ready, set, publicize: which media and why

To be your own best advocate, let the public/community know you exist. Publicize your purpose, publicize your meetings and your accomplishments on a regular basis in all available forms of media that reach the greatest number of people, particularly those you would like as partners and your community. ***Mass media*** refers to all media technologies such as, television, newspapers, film, radio to communicate to the masses. ***Social Media*** refers to websites and other online means of communication used by large groups of people to share information and to develop social and professional networks. Social media networks which include Facebook, MySpace, Linkedin, YouTube and Blogs influence media and directly influence consumers. If you are not currently using social media, you should research their use and make an informed decision as to whether you want to use it to build your presence in the community and globally. You should also consider the following:

#### One-way communication

The type of media you should use depends on the communication you want to establish with your intended audience or participants for your program. One-way communication is when the information being provided does not require a response. If you are advertising a one time or recurring event and you don’t need listeners to respond, mass media is probably sufficient to get your message delivered. Specifically, you want to post flyers in high traffic places where the populations you are trying to reach frequent. This could include grocery stores, churches, schools, fast food restaurants, medical offices or internet cafes. Other means of communications include newsletters, signs, billboards, radio spots, TV spots, taped TV shows, press release, mailed letters, save the date announcements, flyers on car windshields, word of mouth and mass text messaging.

A *Press Release* is an excellent example of a one-way-communication. Write a press release when you have something significant to report, for example if you are just are starting a new initiative, or when you have an event to announce or a report to share with the public, stakeholders and partners. The Press Release should be one page and should answer the questions. Who, what, why, where, when and how?

#### Two-way communication

With two-way communication, information includes response from the recipients of the information. If you are sending a message that requires a response use email, blogs, social media, town hall meetings, or mass text messaging. Another unique two-way communication is to require that prospective attendees pick up a free ticket at defined locations in order to gain access to the event. The number of tickets picked up closes the loop on the two-way-communications; by letting you know how many people to expect. Another alternative could be to give a telephone number or a website address to potential attendees so that they can join your group or simply learn more about your organization. Be prepared to react positively to each and every response in a timely manner. Always remember that you are building a reputation. It only takes one unreturned phone call or one unanswered email for the word to spread like wildfire that your organization doesn’t answer phones or does not return phone calls.

Another two-way communication is a *Press Conference*. A press conference is a tool designed to generate news – in particular, hard news that can advance the cause of your organization. Hard news is defined as a story in the print or electronic media which is timely, significant, prominent, and relevant. There are several things to do to prepare for a *Press Conference*, including**:** Define your message; schedule the date and time of your press conference; Pick the site; Select and train your participants; Contact the media; Follow up with the media; and Develop a press kit. A press kit is a folder of information to give reporters on the background information about your issue or program. The content should be of highest resolution or done with desktop publishing.

### Step 6. Don’t think you have to reinvent the wheel

Finally, one does not have to reinvent the wheel to be successful at initiating a cancer promotion program. Look to success stories in other cancer awareness program. Breast cancer is an excellent example of a cancer advocacy success story [10]. Additional resources for effective cancer control programs are those from the World Health Organization and United States [11-13].

## Conclusions

The content in this article is intended as a general how-to guide for both the beginner and the experienced cancer advocate who want to establish/promote a cancer awareness program. It is not meant to be a comprehensive manual for media engagement. Making a cancer advocacy program visible can be accomplished by using all available existing forms of media to introduce your organization to local, state-wide and to other African advocacy groups across the continent. It is envisioned that an increase in cancer advocacy organizations across the African continent is the catalyst that will have the greatest potential impact on increasing cancer awareness, prevention and ultimately cancer control in Africa.

## Competing interests

The authors declare they have no competing interests.

**Figure 1:**
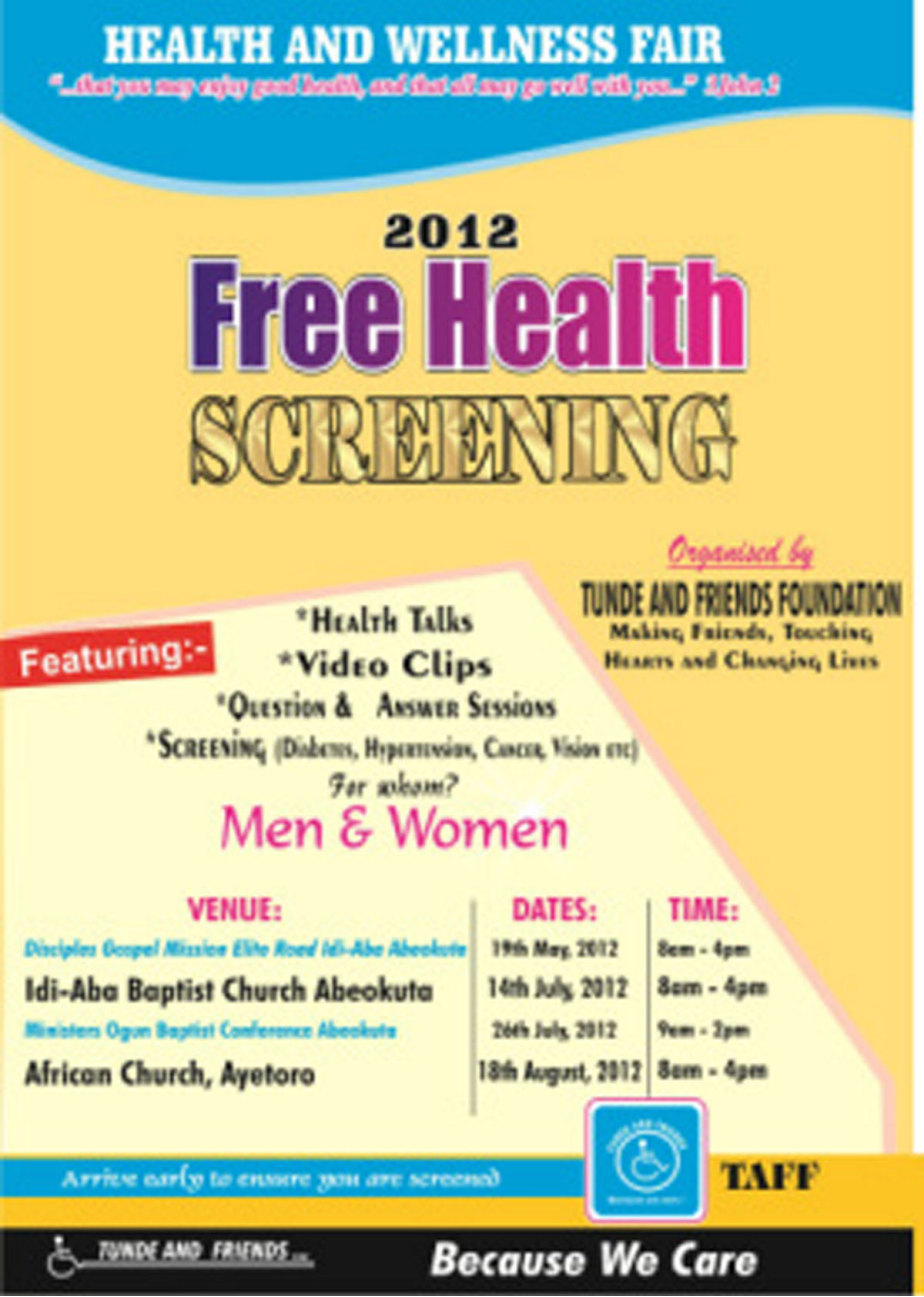
Health and Wellness Fair

**Figure 2:**
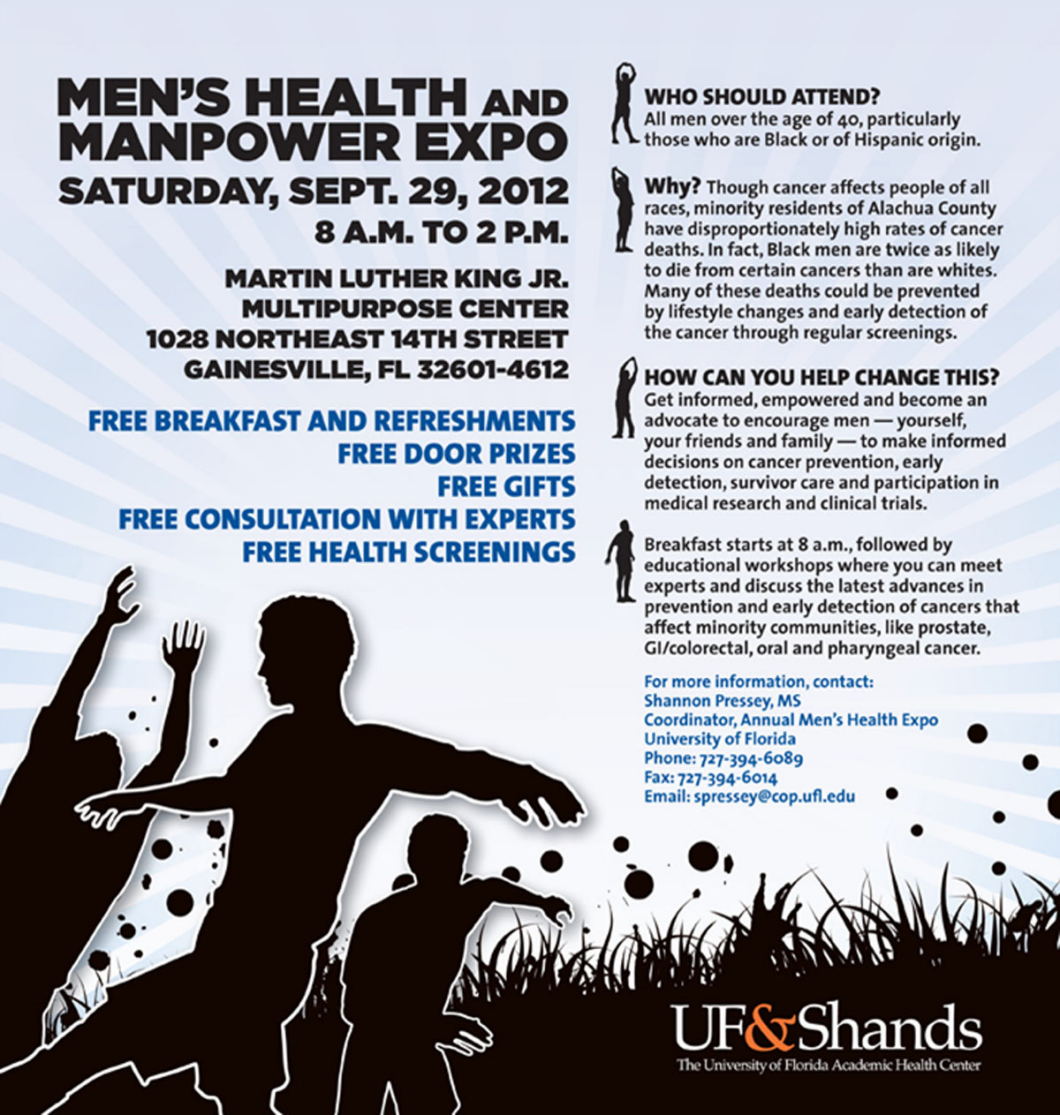
Men’s Health and Manpower Expo 2012

**Figure 3:**
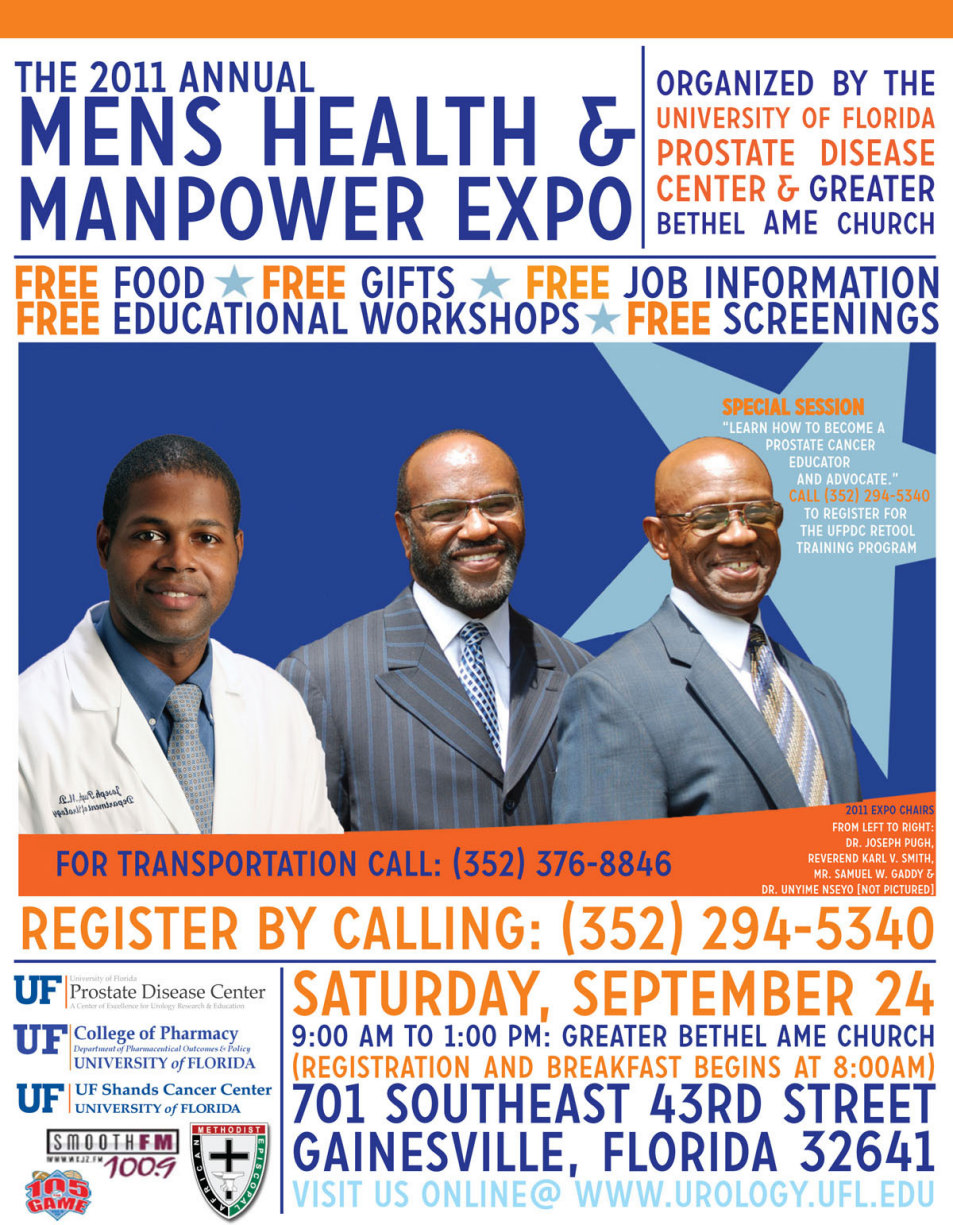
Men’s Health and Manpower Expo 2011

## Supplementary Material

Additional file 1 FAMU Sponsors the Fourth Annual Joshua Hillman Health Fair Click here for file

## Declarations

The authors declare that funding for publication of the article was funded by an award provided by the National Cancer Institute (Award Number R13CA162899).

This article has been published as part of *Infectious Agents and Cancer* Volume 8 Supplement 1, 2013: Selected articles from The International Workshop on Cancer Advocacy for African Countries (CAAC) 2011. The full contents of the supplement are available online at http://www.infectagentscancer.com/supplements/8/S1 The Supplement Editors declare they have no competing interests.
