# Supplementary material for: Advocacy resource: engaging the media and promoting your cancer program in Africa
Source: Infect Agent Cancer. 2013 Jul 15;8(Suppl 1):S5. doi: 10.1186/1750-9378-8-S1-S5 (PMC3716690; doi:10.1186/1750-9378-8-S1-S5)
Supplement: Additional file 1 — FAMU Sponsors the Fourth Annual Joshua Hillman Health Fair [file 1750-9378-8-S1-S5-S1.pdf]

Supplement.

A Sample Press Release: This Fourth Annual Joshua Hillman Health Fair press release informs a local community about a community health fair hosted by the FAMU College of Pharmacy has already occurred. Note that a press release went out before this health to stimulate interest and awareness; the purpose of sending a press release out after the event serves the purpose of updating the local community on the success of the health fair and to let them know that it is an annual event.

Posted: Fri 10:47 PM, Apr 01, 2011

AA
Reporter: FAMU Press Release; Jill Chandler Email
Updated: Tue 6:19 PM, Apr 05, 2011
Back to Florida News

[UPDATE]FAMU Sponsors the Fourth Annual Joshua Hillman Health Fair

Tallahassee, Florida--

More than 400 FAMU students attended the Joshua Hillman Health Fair Tuesday.

The health fair offers free screenings to students and the community. The event is named after Joshua Hillman, a FAMU pharmacy student that passed away because of a medical misdiagnosis.

His mother, Joyce Hillman- Hasan, came to the health fair. She said, "This program is an awesome program... because if it was implemented during the time where he was you know before he passed... it would have been a good thing."

This is the fourth annual health fair. Counseling sessions with doctors, nutritionists, and exercise specialists were also available.

Tallahassee, FL - Florida A&M University's (FAMU) College of Pharmacy and Pharmaceutical Sciences (COPPS) will sponsor the fourth annual Joshua Hillman Health Fair on Tuesday, April 5, from 9 a.m. to 4 p.m. in the New Pharmacy Building. The event is open to the public.

The free screenings include rapid HIV testing; diabetes, osteoporosis/bone density, vision, hearing, cholesterol and much more. Counseling sessions with doctors, nutritionists, and exercise specialists will also be available to discuss the screening results.

The Beta Sigma chapter of Phi Lambda Sigma, a national pharmacy leadership organization housed in the FAMU COPPS, launched the Joshua Hillman Health Initiative to educate and

promote healthy lifestyles, disease prevention and minimize health disparities. The initiative was inspired by Joshua Hillman, a pharmacy student that passed away from a medical misdiagnosis in 2005.

For more information, call (850) 412-7373.

Posted: Fri 10:47 PM, Apr 01, 2011
